# Investigation of Tannins Transformation in *Sanguisorbae Radix* Over Carbonizing by Stir-Frying

**DOI:** 10.3389/fmolb.2022.762224

**Published:** 2022-03-02

**Authors:** Wei Gu, Hao Wang, Man Su, Yiwei Wang, Fei Xu, Qinglian Hu, Xuyi Cai, Jinyun Song, Huangjin Tong, Yuerong Qian, Hongyu Zhao, Jun Chen

**Affiliations:** ^1^ Jiangsu Provincial Engineering Research Center of TCM External Medication Development and Application, Nanjing University of Chinese Medicine, Nanjing, China; ^2^ School of Pharmacy, Nanjing University of Chinese Medicine, Nanjing, China; ^3^ Jiangsu Key Laboratory of Chinese Medicine Processing, Nanjing, China; ^4^ The Key Laboratory of Infection and Immunity of Shandong Province, Department of Pharmacology, School of Basic Medical Sciences, Shandong University, Jinan, China; ^5^ Lianyungang Food and Drug Inspection and Testing Center, Lianyungang, China; ^6^ Department of Clinical Research Center, The Second Hospital of Nanjing, Nanjing University of Chinese Medicine, Nanjing, China; ^7^ Department of Pharmacy, Affiliated Hospital of Integrated Traditional Chinese and Western Medicine, Nanjing University of Chinese Medicine, Nanjing, China

**Keywords:** *Sanguisorba officinalis* L, *Sanguisorbae radix*, carbonizing by stir-frying, processing, tannins

## Abstract

Carbonizing by stir-frying (CSF) is the most common technology in botanical folk medicines to enhance the convergence, hemostasis, and antidiarrheal effects. *Sanguisorbae Radix* (SR), a well-known herbal medicine in China, has extensive therapeutic functions, while charred SR is known as an additional product obtained from SR after CSF. In this study, mass spectrometry was used to investigate the effect of charring on tannins transformation of SR. The findings showed that the content level of tannins in SR decreased significantly after carbonizing process, while their three categories, gallotannins, ellagitannins, and procyanidins, had downward trends in general. Moreover, CSF also induced the polyphenol in SR to release relevant monomers from its origins. Significant amount of hydrolyzable tannins were detected by mass spectrometry, including gallotannins and ellagitannins, suggesting that hydrolysis during CSF yielded gallic and ellagic acid and their derivatives, in addition to sugar moieties. Subsequently, gallic and ellagic acid can further polymerize to form sanguisorbic acid dilactone. The amount of proanthocyanidins, the oligomers of catechin, including procyanidin, procyanidin C2, procyanidin B3, and 3-O-galloylprocyanidin B3, decreased to form catechin and its derivatives, which may further degrade to protocatechualdehyde. Quantitative analysis illustrated that the amount of gallic, pyrogallic, and ellagic acid and methyl gallate, the essential effectors in SR, significantly increased after CSF, with increased ratios of 1.36, 4.28, 10.33, and 4.79, respectively. In contrast, the contents of cathechin and epigallocatechin dropped remarkably with increased ratios of 0.04 and 0.02. Tannins exhibit moderate absorption, while their relevant monomers have a higher bioavailability. Therefore, CSF is proved here to be an effective technique to the release of active monomers from the original polyphenol precursor. This study explored the mechanism by which tannins are transformed upon CSF of SR.

## Introduction

Charcoal is only used as a general adsorbent in western countries, yet carbonized drugs in botanical folk medicines have been reported to have remarkable medical efficacy, particularly their convergence, hemostasis, and antidiarrheal effects. Carbonized botanical medicines have been used for over 2,000 years in China, and 26 types of carbonized drugs and 35 single compound preparations containing charcoal drugs are identified and recorded in the Chinese Pharmacopoeia Commission (2020). Carbonizing by stir-frying (CSF), also named charring processing, is the most used pharmaceutical technology in processing and obtaining carbonized drugs. One of the major principles of traditional charring process is that “carbonizing retains characteristics,” which refers to the partial or total conservation of the innate properties and bioactivity of the drug upon treatment at high temperature. Additionally, the convergence, hemostasis, and antidiarrheal effects can be enhanced by charring (Gao et al., 2020). However, little information is reported on the effects of CSF, especially on how tannins are transformed during herbal charring processes.

*Sanguisorbae Radix* (SR), the root of *Sanguisorba officinalis* L., is a well-known herbal medicine in China, Korea, and Japan. It has extensive therapeutic functions such as hemostatic and astringent properties used in treating bleeding, duodenal ulcers, diarrhea, and chronic intestinal infections. Moreover, the anti-inflammatory, anti-infection, antioxidant, anti-cancer, anti-allergic, anti-wrinkle, and neuroprotective activities of SR extracts or their bioactive constituents have also been previously reported (Chen et al., 2017). Charred SR (CSR) is an additional product obtained after carbonization over stir-frying of SR. Interestingly, after stir-frying, the bioactivities in terms of hemostatic, burn wound healing, and antibacterial effects were greatly enhanced compared to those of raw SR (Ding et al., 1995; Guo et al., 2001; Gu et al., 2020; Gu et al., 2021). Numerous studies had showed a better hemostatic effect (Jia et al., 1992; Guo et al., 2001; Yu et al., 2014; Zhang et al., 2017) as well as a better bacteriostatic effect (Ding et al., 1995; Gu et al., 2020; Gu et al., 2021) of CSR compared to that of SR. Besides, among the different processed products of SR, CSR is the best in treating burn wound (Lei, 2021). CSR's promoted bioactivity may be due to the changes in composition after the charring process, which could play an important role in the *in vivo* pharmacodynamics. However, to the best of our knowledge, there is very limited research and data on SR processing.

The bioactive components of SR include tannin, flavone, saponin, and steroid compounds (Xia et al., 2009; Wu et al., 2014). Tannins, astringent and bitter-tasting plant polyphenols, are the highest bioactive constituents in SR, with a reported percentage up to 12–17% (Cai, 2019). Tannins possess extensive pharmacological activities such as anti-oxidant, anti-cancer, antibacterial, anti-inflammatory, anti-diabetic, and anti-microbial effects (Fernando et al., 2013; Vázquez-Fresno et al., 2016). Therefore, tannins are believed to be the main active ingredient in SR and are selected to be the quality control index for SR and CSR based on theChinese Pharmacopoeia Commission (2020). The content level of tannins in SR and CSR should be above 8% and 2%, respectively. However, tannins exhibit very high variability in their structures. Hundreds of unique molecules were detected in SR, with molecular weights ranging from 500 to 3,000 Da (Bele et al., 2010). Previous studies only focused on the content changes of several index components before and after charring of SR, thus lacking evidence supporting the carbonizing processing mechanisms. Therefore, the aim of this study is to investigate the structure changes of tannins in SR during carbonizing and to further explain the processing effect on their pharmacodynamics in future studies.

## Materials and Methods

### Reagents

Gallic acid (GA) (lot number: C13O9C72105) and methyl gallate (MG) (lot number: AN1127SA14) were obtained from Shanghai Yuanye Bio-Technology Co., Ltd. (Shanghai, China). Pyrogallic acid (PA) (lot number: HA0820KA14) and catechin (lot number: P02A9F57645) were purchased from China National Institutes for Drug Control (Beijing, China). Epigallocatechin (EGC) (lot number: 1806125) was obtained from Shanghai Aladdin Bio-Chem Technology Co., Ltd. (Shanghai, China). Protocatechnic aldehyde (lot number: YECQ20190819) was purchased from Nanjing Spring & Autumn Biotech Co. Ltd. (Jiangsu, China). Ethyl gallate (EG) (lot number: 191127) was offered from Nanjing Jin Yibai Biological Technology Co. Ltd. (Jiangsu, China), and ellagic acid (EA) (lot number: 817C024) was obtained from Sichuan Victory Biological Technology Co., Ltd. (Sichuan, China). Liquid chromatography-mass spectrometry (LC-MS) grade methanol (lot number: 20025141) and acetonitrile (lot number: 19105068) was purchased from E. Merck (Merck, Darmstadt, Germany). Formic acid with a purity of 99% and of LC-MS grade was obtained from Anaqua Chemical Supply (ACS, Houston, United States).

### Preparation of *Sanguisorbae Radix* and Charred *Sanguisorbae Radix*

SR (lot number: 20160240) was collected from Suzhou Tianling Chinese Herbal Medicine Co. Ltd. (Jiangsu, China). Samples were randomly divided into two groups. One group was processed into Charred *Sanguisorbae Radix* (CSR) *via* an automatic frying machine in accordance with the Chinese Pharmacopoeia Commission (2020) (model: MSDC-5, Changzhou Jintan Meisi Machinery Co., Ltd.). The experimental steps are as follows: The temperature and the rotating speed of the automatic frying machine was set at 230°C and 26 rpm/min, respectively. After the frying machine was heated up to the set temperature, about 100 g SR was added into the machine. Seven minutes later, CSR was obtained, with a charcoal-colored surface and an ustulate interior area.

### Preparation of Extraction of *Sanguisorbae Radix* and Charred *Sanguisorbae Radix*

A certain amount of SR and CSR powder was immersed into 75% ethanol for 12 h at 50°C. After that, their ingredients were extracted *via* ultrasonic-assisted extraction for 1 h. After vacuum filtration, the extracts were evaporated under water vacuum at 40°C using a rotary evaporator. The contents of total tannins in the SR/CSR extracts were measured and were 18.65 ± 0.06% w/w and 7.20 ± 0.07% w/w, respectively, using the methodology designed for tannins [general rule 2202 of theChinese Pharmacopoeia Commission (2020)].

## Qualitative Analysis and Relative Quantification by Ultra Performance Liquid Chromatography-Quadrupole/Time-Of-Flight-Tandem Mass Spectroscopy

### Chromatographic Analysis Conditions

Samples were injected into an Agilent 1260 Infinity LC System, connected to an Agilent 6540 accurate mass quadrupole/time-of-flight (Q/TOF) LC-MS system (Santa Clara, CA, United States).

*High-performance liquid chromatography (HPLC) conditions:* Agilent Poroshell 120 EC-C18 (2.1 mm × 150 mm, 2.5 μm, Waters, United States) was used for chromatographic separation. The binary mobile phase was composed of acetonitrile (A) and water containing 0.2% (v/v) formic acid (B) at a flow rate of 0.3 ml/min. The linear gradient elution system was conducted as follows: 0–27 min, 5%–70% A; 27–30.3 min, 70%–50% A; 30.3–40 min, 50%–50%A; 40–40.5 min, 50%–95% A; 40.5–50 min, 95%–5% A; and 50–62 min, 5% A. The re-equilibration time was set to 4 min with a total running time of 62 min. The column compartment was set at 30°C, while the auto-sampler trial was kept at 15°C. After each injection (1 μl), the needle was washed with mixtures of acetonitrile and water.

*MS conditions:* The capillary voltage and capillary outlet voltage were 3.5 kV and 100 V, respectively, and atomization gas was 241.3 kPa. Dry gas temperature and flow were set at 300°C and 10 L/min. The mass scanning was controlled in the range of m/z 100–600.

*Tandem mass spectroscopy (MS/MS) conditions:* The spectra were conducted under positive and negative ion types. The capillary voltage was 5,500 V at positive ion type and −4,500 V at negative ion type, and desolvation gas temperature was 550°C. The curtain gas was 241.3 kPa, and both nebulizer and drying gas were 379.2 kPa. Nitrogen was used as source gas with purity more than 95% and collision gas with purity over 99.999%.

### Compound Identification

Agilent MassHunter Qualitative Analysis (version B.06.00 SP1) software (Santa Clara, CA, United States) was used for processing MS and Auto MS/MS data acquired using ultra-performance liquid chromatography (UPLC)-Q/TOF-MS/MS. The accurate mass MS data were processed using the tool “Find by Molecular Feature” to export the compounds to Agilent Mass Profiler Professional (MPP) software (Santa Clara, CA, United States). Aiming to remove the molecular features from the background, the data acquired from each fraction were background-subtracted using the blank data. By reviewing domestic and abroad reference and databases like “Chemspider” and “TCMSP,” a local database of 140 chemical components of SR and CSR was established (Xia, 2010; Xu et al., 2018).

### Data Analysis

All data were normalized by peak area to obtain the normalized intensities (NIs) of chromatographic peaks, which were used to predict the relative contents of corresponding compounds. The comparison between two samples was expressed by FoldChange (FC), which was calculated using formula (*). FC ≥ 1.5 indicates the increased content of compound after charring; FC ≥ 5 means a significant increase; FC ≤ 0.67 means a decrease, and FC ≤ 0.2 was determined as a significant decrease.

$$\mathrm{FC}=\frac{\mathrm{The NIs of chromatographic peak in CSR}}{\mathrm{The NIs of chromatographic peak in SR}}\;\left(\text{*}\right)$$

## Accurate Quantification by Ultra-High Performance Liquid Chromatography-MS/MS

### Chromatographic Analysis Conditions

All samples were analyzed in triplicate using the quadrupole mass spectrometer, model Triple Quad 5500 (ABSCIEX, FosterCity, CA), which was controlled by Analyst^®^ 1.6 software. The mass spectrometer was directly coupled with an ultra-high performance liquid chromatography (UHPLC) system from Shimadzu Corporation (Shimadzu, Kyoto, Japan), consisting of a SIL-30AC autosampler, a CBM-20A Lite controller, a DGU-20A5 degasser, a CTO-20A column heater, and two LC-30AD pumps.

*HPLC conditions:* Agilent Extend-C18 column (100 mm × 2.1 mm, 1.8 μm) was used for chromatographic separation. The mobile phase consisted of 0.1% formic acid-water (A) and acetonitrile (B) at a flow rate of 0.2 ml/min, with the gradient elution system set as follows: 0–1 min, 5–50% B; 1–5 min, 50–95% B; 5–6 min, 95% B; 6–7 min, 95–5% B, 7–8 min, 5% B. The sample injection volume was set at 1 μl, and the column oven temperature was kept at 30°C.

*MS conditions:* Mass spectrometric detection was conducted in the negative mode with an electrospray ionization (ESI) source. Ion spray voltage and detection temperature were −4,500 V and 550°C, respectively. Curtain gas (CUR), ion source gas 1, and ion source gas 2 (GAS2) were respectively set at 35, 55, and 55 psi for all the analytes. The quantification was acquired using a multiple reaction monitoring (MRM) mode. The compound-dependent parameters of eight analytes are listed in Table 1. The entrance potential (EP) and the collision exit potential (CXP) were set at −10.0 and −17.0 V, respectively.

**TABLE 1:** MS/MS parameter of eight analytes.

Compounds	Polarity	MW (Da)	Precursor-product ion transition (m/z)		Declustering potential (Volts)	Collision energy (Volts)	Retention time/min
			Q1	MS^2^			
Gallic acid	[M-H]^-^	170.12	169.1	125.0	−40.98	−20.25	1.16
				79.0	−20.41	−26.12	
Pyrogallic acid	[M-H]^-^	126.11	124.9	79.0	−46.36	−24.87	1.51
				69.1	−84.36	−22.24	
Epigallocatechin	[M-H]^-^	306.27	305.1	125.0	−80.33	−24.04	2.06
				137.0	−49.96	−30.13	
Catechin	[M-H]^-^	290.27	289.1	245.1	−22.9	−19.02	2.26
				203.0	−17.18	−25.71	
Protocatechnic aldehyde	[M-H]^-^	138.12	137.1	108.1	−69.47	−31.85	2.34
				91.9	−64.23	−30.66	
Methyl gallate	[M-H]^-^	184.15	183.1	124.1	−39.54	−22.3	2.40
				168.0	−69.64	−19.4	
Ellagic acid	[M-H]^-^	302.28	301.2	284.1	−86.86	−39.11	3.07
				145.1	−59.11	−47.71	
Ethyl gallate	[M-H]^-^	198.17	197.0	123.7	−53.6	−24.02	3.28
				168.9	−44.55	−18.72	

### Stability

The stock solutions of eight standards (Table 1) were individually prepared with methanol and stored at 4°C. Each standard solution was injected at 0, 4, 8, 12, 16, and 24 h to assess the stability of the eight analytes. All samples were analyzed in triplicate.

### Recovery

Recovery was performed according to the latest guidance edited in 2018 by analyzing the herbal extraction after adding a mixture of eight standards at low, medium, and high levels, with three repeats per concentration.

### Standard Curve

Combined standard working solutions at a series of different concentrations were prepared by further diluting the mixture of eight standards with methanol. The standard curve was established by plotting peak area to the nominal concentration of eight analytes with a weighted (1/x^2^) least-squares linear regression.

## Results and Discussion

### Qualitative Analysis and Relative Quantification by UPLC-Q/TOF-MS/MS

**FIGURE 1:**
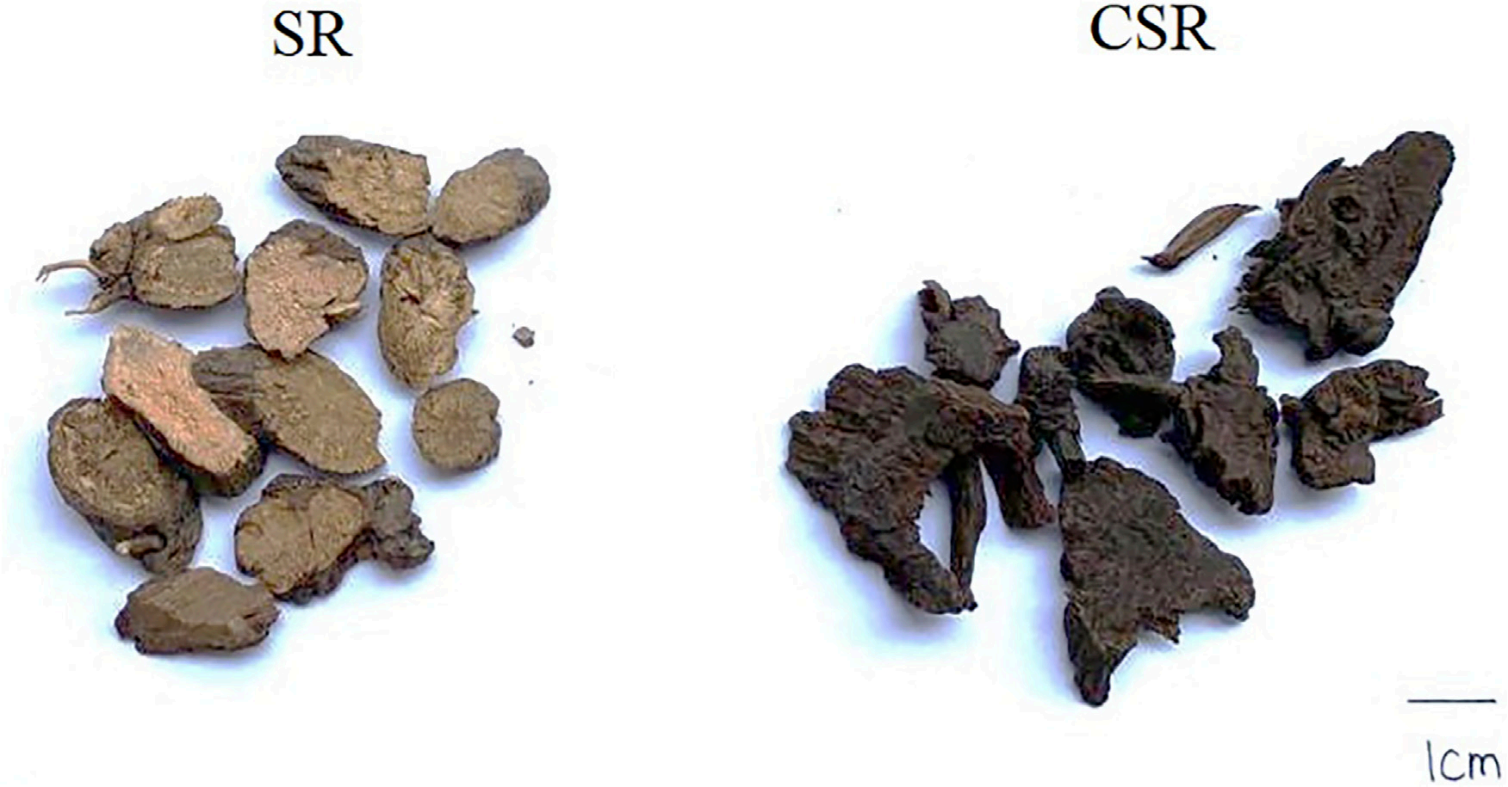
The representative images of SR and CSR.

The representative image of SR and CSR is shown in Figure 1. A local database of 140 chemical components of SR and CSR was established firstly. Among the 140 compounds, 56 compounds were identified as all fractions in SR/CSR using accurate mass precursor and fragment ion information. Mass spectrum of five compounds, including GA, EA, catechin, MG, and EG had also been identified based on their relevant standards.

**FIGURE 2:**
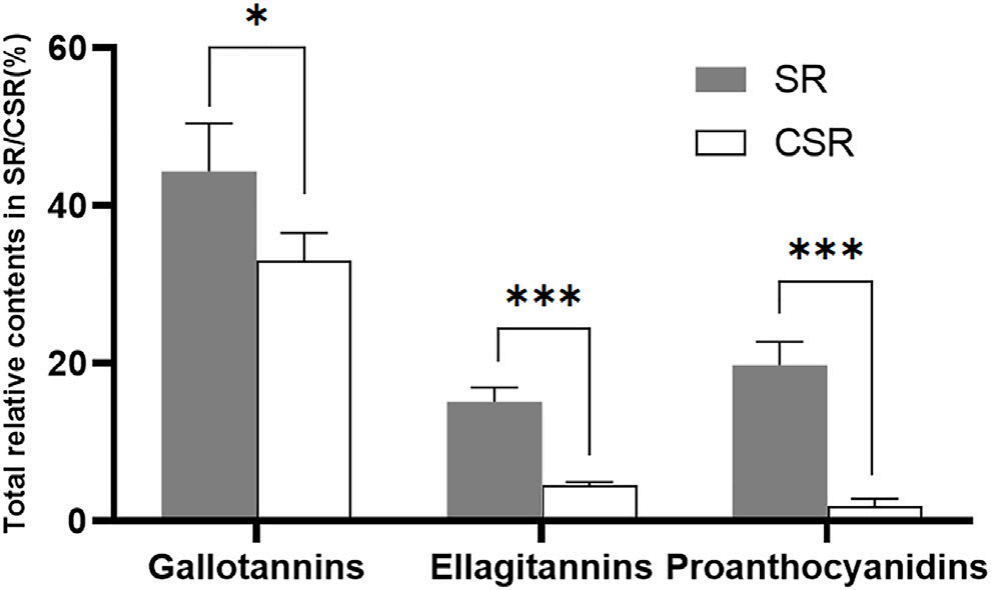
The relative contents calculated by peak area normalization of all tannins in SR/CSR(*n* = 3) (Note: *p < 0.05, ***p < 0.001).

Tannins are water-soluble polyphenols with a molecular weight ranging from 120 to 3,000 Da. Attributed to the phenolic cores, hydrolyzable tannins (HTs) and proanthocyanidins (PCs) were determined to be the active compounds (Salminen et al., 2011; Sathya et al., 2017). HTs are subdivided into gallotannins and ellagitannins, containing a core of glucose with GA, or with EA, respectively. They can be hydrolyzed to yield phenolic acids and carbohydrate in mild acids or mild bases environment, which normally does not occur in nature. In the present study, 39 of 56 compounds were determined to be tannins in the extracts of SR/CSR. Gallotannins, ellagitannins, and PCs contained 21, 9, and 9 compounds, respectively (Supplementary Table S1). The recorded molecular weights were in the range of 120–1,000 Da. The relative content of each compound was calculated by chromatographic peak area normalization of all tannins in SR/CSR. As shown in Figure 2, significant drops (*p* < 0.05) were observed in the contents of gallotannins, ellagitannins, and PCs in SR after CSF. This finding was consistent with the decrease of total amount of tannins in SR. The contents of total tannins in the SR/CSR extracts were 18.65 ± 0.06% w/w and 7.20 ± 0.07% w/w, respectively.

### Structure Transformation of Gallotannins in SR During Carbonizing by Stir-Frying

**FIGURE 3:**
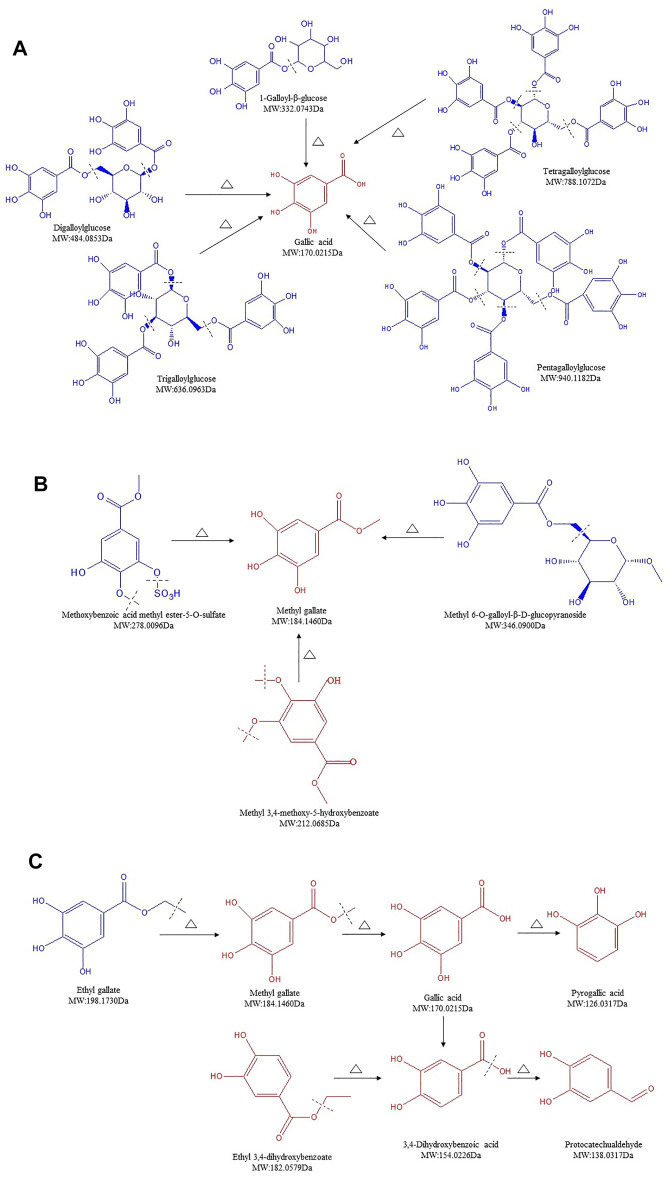
Structure transformation of gallotannins in *Sanguisorbae Radix* during carbonizing by stir-frying (Note: The compound identified in blue means the relative content in SR decreased during carbonizing, while in red indicates an increase).

Gallotannins are considered as the simplest form of HTs, which upon hydrolysis yields GA, in addition to sugar moieties. Furthermore, galloylation yields di-, tri-, tetra-, penta-, hexa-, hepta-, and octagalloylglucoses (Sieniawska and Baj, 2017). Twenty-one gallotannins were identified in SR/CSR (Supplementary Table S1, lines 1–21), and among them, 8 compounds' contents showed an upward trend, while 10 compounds showed a downward trend. In general, the contents of glycosides as well as methoxybenzoic acid methyl ester-5-O-sulfate gradually or significantly decreased, including 1-galloyl-glucose, digalloylglucose, trigalloylglucose, tetragalloylglucose, pentagalloylglucose, and methyl 6-O-galloyl-β-D-glucopyranoside. However, the amount of GA, PA, and MG increased remarkably. The hydroxyl group of glucose was proved to form an ester bond with the carboxyl group of GA or ether bond with the phenolic hydroxyl group, which are the unique characteristic components of SR (Xu et al., 2018). A recent study also confirmed that the hydrolysis of HTs in *Phyllanthus emblica* L. during the heating reflux extraction process (e.g., chebulagic acid, corilagin, and hydrolysis) may result in an increased content of GA (Huang et al., 2019). Therefore, the hypothesis is that galloylglucosides can be hydrolyzed into GA and glucose during processing (Figure 3A). MG is the terminal hydrolysate of methoxybenzoic acid methyl ester-5-O-sulfate, methyl 6-O-galloyl-α-D-glucopyranosid, and methoxybenzoic acid methyl ester-5-O-sulfate (Figure 3B), while EG can be further transformed into MG which can be further decarboxylated to form PA (Zhang, 2013) (Figure 3C).

Previous studies showed that the content of GA in SR can fluctuate, with an increase followed by a gradual reduction depending on the degree of processing (Zhao et al., 2018). Our findings (Figure 3) revealed the same phenomenon regarding SR due to the cleavage of the glycoside bonds or ester bonds of HTs in SR, thus releasing free GA. It was found that GA can further form PA under secondary processing, resulting in a significantly increased content of PA. Although EG is also the hydrolysate of glycosides, it can be further transformed into MG, leading to a reduced content of EG and a significant production of MG (Supplementary Table S1). These results were in agreement with previous studies showing a comparable effect of CSF on pomegranate rind (Cui et al., 2010; Zhou et al., 2014).

### Structure Transformation of Ellagitannins in SR During Carbonizing by Stir-Frying

Ellagitannins are another form of HTs, which, in addition to sugar moieties, yield EA upon hydrolysis (Sieniawska and Baj, 2017). Ellagitannins can exist as monomeric, dimeric, oligomeric, or C-glycosidic forms (Takuo and Hideyuki, 2011). There were nine ellagitannins detected in SR/CSR (Supplementary Table S1, lines 22–30). The content of five compounds showed an upward trend, whereas the other four compounds showed an opposite downward trend. Compared to gallotannins, the contents of macromolecular compounds such as 3,3′,4′-trimethylellagic acid-4-O-sulphate, 3,4′-dimethylellagic acid-4-O-sulphate, 3,3′,4-tri-O-methylellagic acid-4-O-β-D-xylopyranoside, and 3,4′-di-O-methylellagic acid-4-O-β-D-xylopyranoside were all found to decrease greatly, whereas a significant increase of their monomers, 3,3′,4′-tri-O-methylellagic acid, 3,4′-O-dimethylellagic acid, and EA, was observed.

**FIGURE 4:**
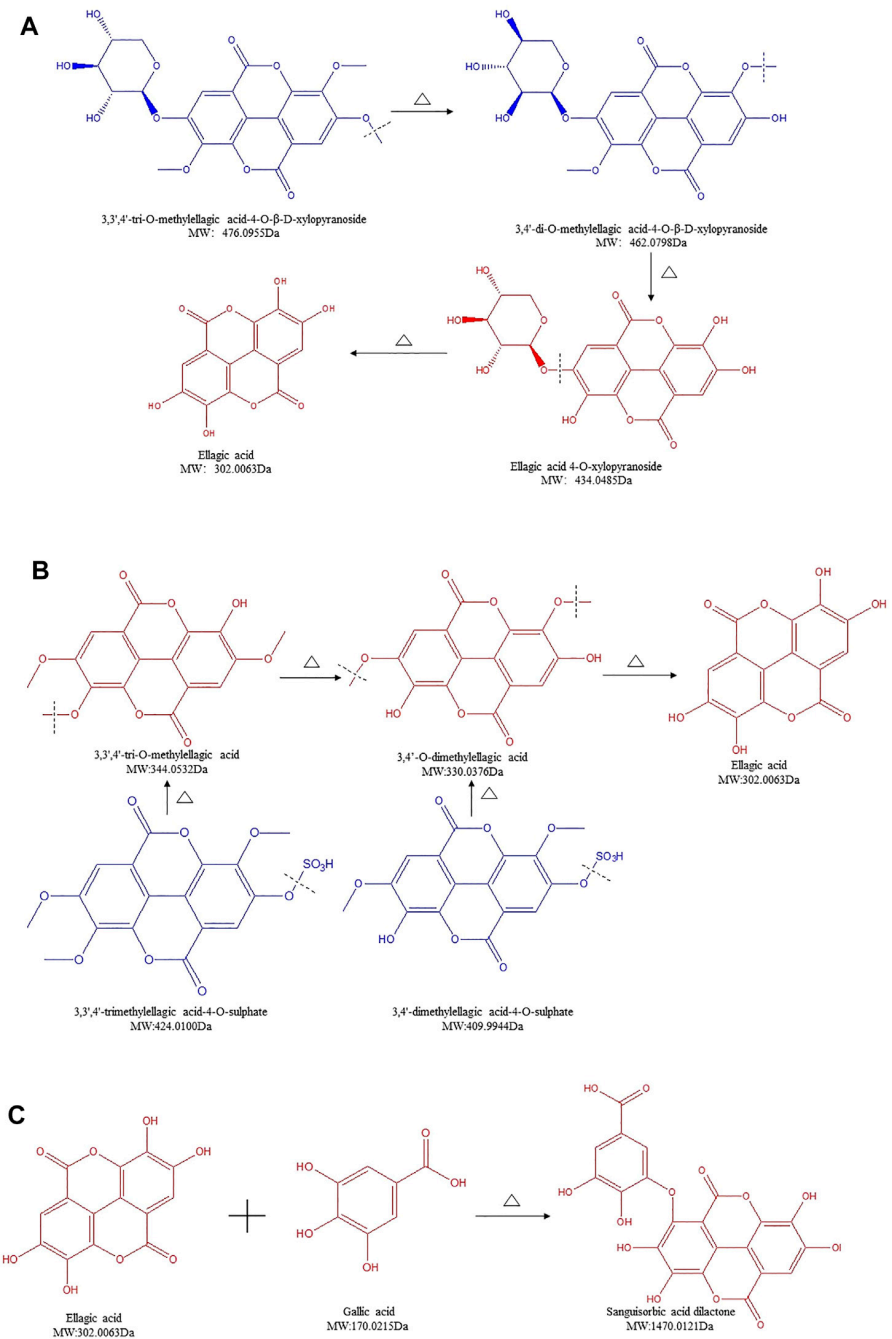
Structure transformation of ellagitannins in *Sanguisorbae Radix* during carbonizing by stir-frying (Note: The compound identified in blue means the relative content in SR decreased during carbonizing, while in red indicates an increase.)

Glycosidic bonds of HTs can be easily affected at high temperature. With a great molecular weight, it tends to break easily. Therefore, we hypothesize that the macromolecular ellagitannins in SR were hydrolyzed into EA, and their derivatives after CSF. 3,3′,4′-tri-O-methylellagic acid-4-O-β-D-xylopyranoside can be converted to 3,4′-di-O-methylellagic acid-4-O-β-D-xylopyranoside, followed by a secondary reaction to form ellagic acid 4-O-xylopyranoside and ultimately resulting in EA (Figure 4A). This chemical reaction can be used to explain this interesting phenomenon, which is different from those of other glycosides. The amount of ellagic acid 4-O-xylopyranoside increased highly. By removing the sulfate bonds, 3,3′,4′-trimethylellagic acid-4-O-sulphate and 3,4′-dimethylellagic acid-4-O-sulphate were respectively transformed into 3,3′,4′-tri-O-methylellagic acid and 3,4′-O-dimethylellagic acid then gradually further converted into EA (Figure 4B). Findings shown in Figures 4A, B led to a rapid increase of the end-product EA's content. Our previous findings support these data, as the amount of EA in CSR is positively correlated with the degree of processing, which is also in accordance with the results obtained with pomegranate rind, a traditional Chinese medicine (TCM) rich in EA (Dai et al., 2009; Cui et al., 2010; Zhou et al., 2014). Furthermore, the content of sanguisorbic acid dilactone in SR increased significantly after processing. Since both GA and EA's amount increased, it is speculated that they were polymerized to form sanguisorbic acid dilactone (Figure 4C).

### Structure Transformation of Proanthocyanidins in SR During Carbonizing by Stir-Frying

PCs, also known as flavan-3-ol polymers, are considered as an important part of plant polyphenols (Rue et al., 2018). Oligomeric and polymeric forms of PCs, composed of monomeric flavan-3-ol units such as catechin, acquire highly complex chemical structures and high molecular weights ranging from 1,000 to 20,000 Da (Deprez et al., 2001). Unlike HTs, PCs can be only depolymerized by strong acidic or oxidative hydrolysis (Mcsweeney et al., 2001). In our analysis, nine PCs were detected in SR/CSR (Supplementary Table S1, lines 31–39). Surprisingly, most of these contents decreased over 10 times after processing, regardless of their molecular weights.

**FIGURE 5:**
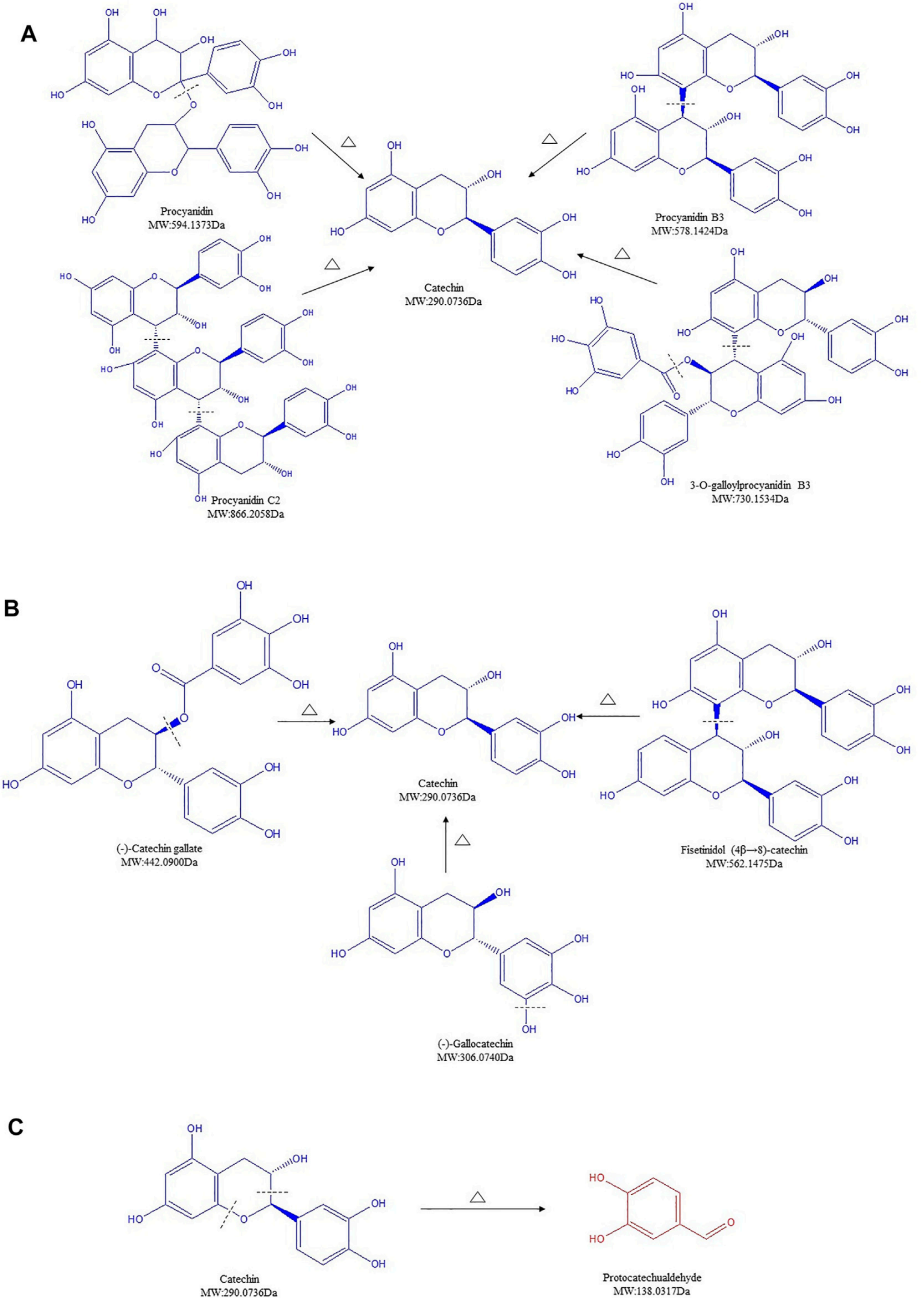
Structure transformation of proanthocyanidins in *Sanguisorbae Radix* during carbonizing by stir-frying (Note: The compound identified in blue means the relative content in SR decreased during carbonizing, while in red indicates an increase.)

Procyanidin, procyanidin C2, procyanidin B3, and 3-O-galloylprocyanidin B3, with an initial high total content of 9.396%, decreased remarkably to 0.689% in CSR. The structure of PCs can be destroyed when the temperature exceeds 100°C, together with a start of hydrolysis and GA removal reaction. Studies on the hydrolysis of highly polymerized PCs further proved that the connecting bonds in PCs polymer units are easily broken in an acidic environment (Guanhong, 2006). When the carbonizing temperature of SR was above 200°C, the extraction condition of SR/CSR was in an acidic environment (extraction pH of both SR and CSR was 4.2). Thus, the significant decrease of PCs' content after processing could be partly due to hydrolysis (Figure 5A).

Besides, catechins contain ester catechins and non-galloylated catechins. The molecular structures of the former compounds bind one or two more galloyl groups compared to those of the latter compounds. Epicatechin gallate (ECG) and catechin are representative compounds of ester catechins and non-galloylated catechins, respectively. It has been proved that non-galloylated catechins can produce GA through high temperature treatment (Guanhong, 2006). After heating, ECG can undergo a degradation, isomerization, and GA removal reaction. Among these three reactions, degradation is highly likely to be the dominant reaction compared to isomerization, which has the lowest chase counting for ECG. Within increased treatment temperature, the reaction rates increased (Wu et al., 2010; Wu, 2011). Because the processing temperature of SR was above 200°C, degradation, isomerization, and GA removal might all be involved in the formation of catechin. Our data suggested that ECG can be transformed to catechin by removal of GA (Figure 5B). Meanwhile, (-)-gallocatechin or fisetinidol (4β→8)-catechin may degrade to form catechin by the removal of a hydroxyl or fisetinol group, as seen in the MS^2^ behavior (Figure 5B).

At last, catechin, which was found to have the highest content in the SR, remarkably decreased after charring (Supplementary Table S1). Such rapid reduction of catechin was also observed over the processing of Rhei Radix Et Rhizoma, Moutan Cortex, and Ploygoni multiflora Radix (Wang et al., 2010; Huang et al., 2016; Huang, 2018). A previous study showed that after light induction, the C-O bond of C ring of catechin breaks off, leading to an open structure of heterocyclic and formation of two radicals: radical A ([M-H]^-^ m/z 137) and radical B ([M-H]^-^ m/z 289). Compound P1 ([M-H]^-^ m/z 137) was formed after electron transfer (Nie, 2017). Here, the content of compound ([M-H]^-^ m/z 137.0251), initially identified as protocatechualdehyde (PCA) increased 19 times after processing, rising from 0.105% to 2.084% (Supplementary Table S1, line 40). The increased compound is expected to be the final compound via the conversion of catechin to compound P1 during processing, which may partially explain the significant decline of catechin's content (Figure 5C).

### Accurate Quantification by UHPLC-MS/MS

**FIGURE 6:**
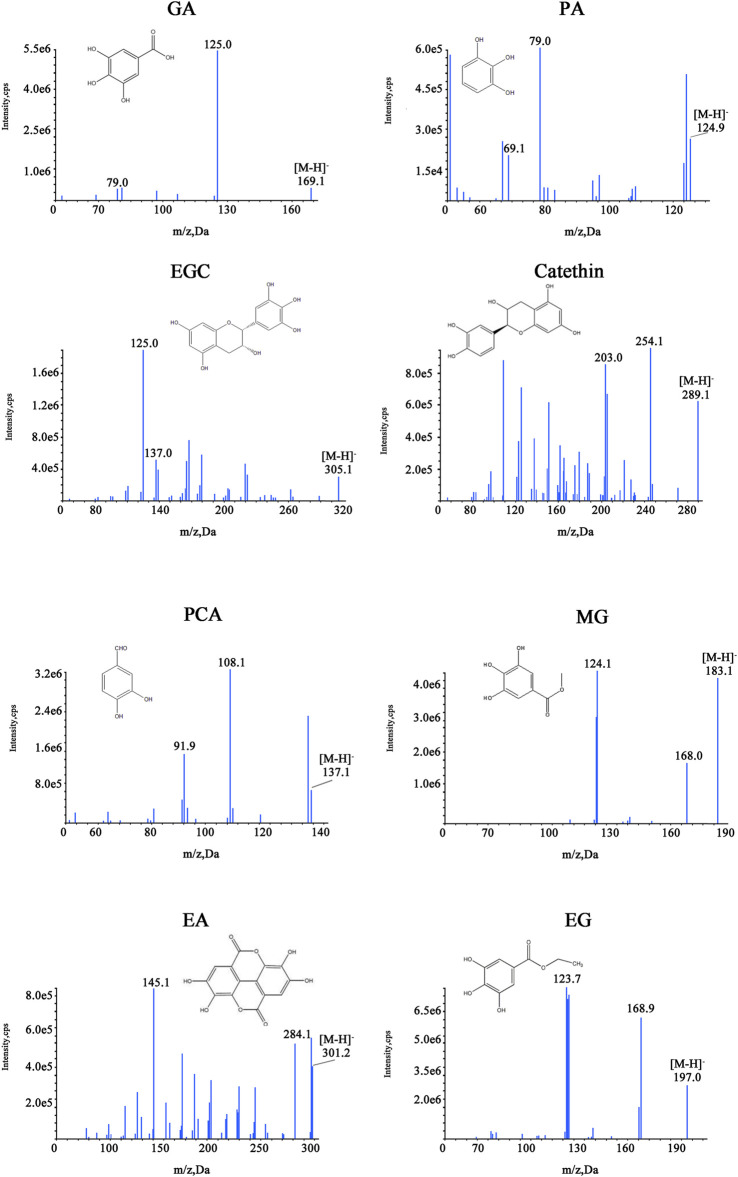
Chemical structures and MS/MS scan spectrums.

**FIGURE 7:**
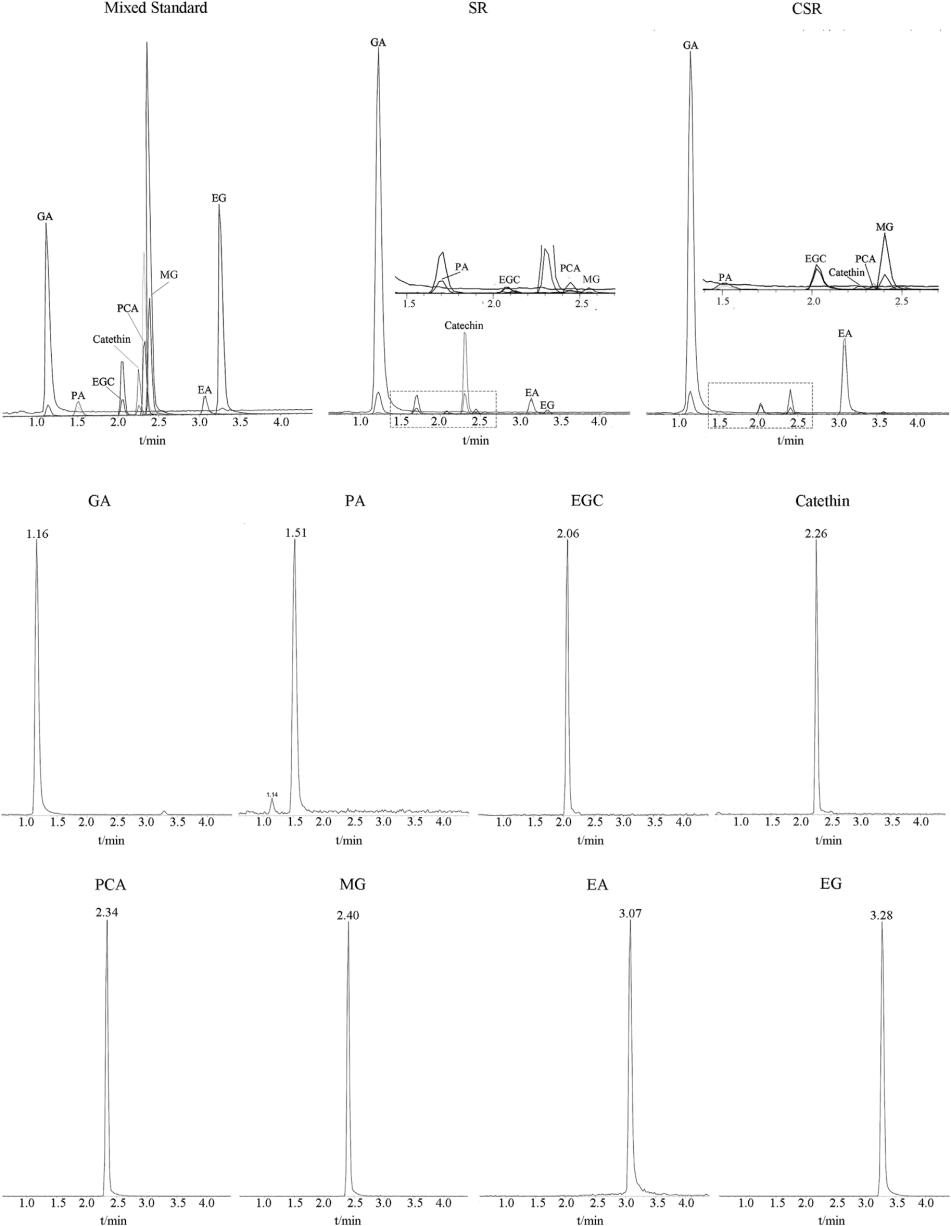
MRM chromatograms of eight analytes in Mixed standard solution, SR solution and CSR solution.

The MS/MS scan spectra of the eight analytes with their chemical structures are exhibited in Figure 6. The MRM chromatograms of these compounds in the combined standard, SR, and CSR solutions are shown in Figure 7.

The stability and recovery results of the eight analytes are summarized in Table 2. The relative standard deviations (RSDs) calculated by the concentrations were found to be less than 2%, indicating that the eight analytes were stable within 24 h. Meanwhile, the recovery of the analytes ranged within 96.70–114.77%. These results showed an acceptable recovery of the developed method. Besides, the correlation coefficients, regression equation, and linear ranges for the eight analytes were measured and are displayed in Table 3. All correlation coefficients were higher than 0.9953, which shows good linearities.

**TABLE 2:** Stability and recovery of the eight analytes (*n* = 3).

Compounds	Stability	Recovery					
		High concentration		Medium concentration		Low concentration	
	RSD%	Mean %	RSD %	Mean %	RSD %	Mean %	RSD %
Gallic acid	1.25	104.73	1.85	98.23	1.21	100.55	1.88
Pyrogallic acid	1.59	103.24	1.54	103.43	1.99	102.73	1.05
Protocatechnic aldehyde	1.66	105.82	1.76	108.63	2.78	117.18	6.54
Catechin	0.96	96.70	3.47	100.97	8.97	107.59	10.39
Methyl gallate	1.68	105.14	2.52	107.39	2.14	114.77	5.28
Ethyl gallate	1.85	104.43	3.42	104.53	3.33	107.87	1.76
Ellagic acid	1.82	97.90	2.84	99.08	1.29	97.47	3.62
Epigallocatechin	1.90	98.75	5.77	99.96	5.19	104.75	4.30

**TABLE 3:** Regression equation, correlation coefficients, and linearity ranges for the eight analytes.

Compounds	Linear range (ng/ml)	Regression equation	R
Gallic acid	9.6–288.0	y = 57965x + 433072	0.9995
Pyrogallic acid	11.0–220.0	y = 4761.1x + 9548.5	0.9999
Protocatechnic aldehyde	10.0–201.0	y = 32231x + 331924	0.9953
Catechin	5.6–225.0	y = 8608.1x + 14476	0.9999
Methyl gallate	10.6–212.0	y = 23157x + 115120	0.9997
Ethyl gallate	10.7–213.0	y = 48759x + 155259	0.9998
Ellagic acid	10.8–215.0	y = 5277.8x + 45490	0.9995
Epigallocatechin	11.2–223.0	y = 13198x − 3383.7	1.0000

y, the peak area of analytes; x, the concentration of analytes.

As shown in Table 4, the quantitative results of the eight analytes' trend in SR/CSR was found to be the same as the data calculated by peak area normalization in UPLC-Q/TOF-MS/MS. The contents of catechin (17.43 ± 0.23 mg/g), GA (12.49 ± 0.70 mg/g), EA (6.89 ± 0.40 mg/g), and EGC (2.29 ± 0.51 mg/g) in SR were very high., whereas those of catechin and EGC decreased to an extremely low level after CSF. In addition, two hydrolysis end-products of glycosides increased greatly: GA increased by 36%, and EA increased by more than 10 times.

**TABLE 4:** Quantitative results of the eight analytes in the extracts of SR and CSR (*n* = 3).

Compounds	Content (mg/g)		Increased ratio after charring	Trend after charring	
	SR	CSR		Calculated by quantitative results	Calculated by peak area normalization
Epigallocatechin	2.29 ± 0.51	0.04 ± 0.01	0.02	↓↓	↓↓
Catechin	17.43 ± 0.23	0.76 ± 0.09	0.04	↓↓	↓↓
Ethyl gallate	0.18 ± 0.00	0.09 ± 0.00	0.50	↓	↓
Gallic acid	12.49 ± 0.70	16.96 ± 0.43	1.36	↑	↑
Pyrogallic acid	0.60 ± 0.02	2.57 ± 0.08	4.28	↑↑	↑↑
Methyl gallate	0.28 ± 0.01	1.34 ± 0.02	4.79	↑↑	↑↑
Ellagic acid	6.89 ± 0.40	71.18 ± 1.94	10.33	↑↑	↑↑
Protocatechualdehyde	0.00 ± 0.00	0.08 ± 0.00	∞	↑↑	↑↑

PA and MG with a low content originally increased by more than 4 times after processing. As GA, PA, EA, and MG are the essential effectors in SR/CSR, it can be expected that the contents of active components increased significantly after processing in parallel with the reduction of total tannins and most components.

In general, the measured total tannin contents in the SR/CSR extracts were 18.65 ± 0.06% and 7.20 ± 0.07%, respectively. There were 39 tannins detected in SR/CSR, with molecular weights ranging from 120 to 1,000 Da. Gallotannins, ellagitannins, and PCs showed significant downward tendency in general after CSF (*p* < 0.05). This is consistent with previous studies showing the decrease in contents of most components, as well as the increase of small amounts of components after CSF of TCM (Gao et al., 2020).

Chemically, CSF of SR was a process that releases the monomers from polyphenol precursor. The main possible chemical reactions were summarized as follows: ① A high amount of HTs, including gallotannins and ellagitannins, upon hydrolysis yielded GA and EA, respectively, in addition to sugar moieties. GA could further be decarboxylated to form PA. GA and EA polymerized to form sanguisorbic acid dilactone. ② PCs, including procyanidin, procyanidin B3, procyanidin C2, and 3-O-galloylprocyanidin B3, oligomers of catechin, were destroyed above 200°C to ultimately form catechin and its derivatives, which might further degrade to PCA.

Analytes' quantitative results further confirm that the transformation trend of tannins of SR during carbonizing is based on the calculation *via* peak area normalization in UPLC-Q/TOF-MS/MS. Since GA (increased ratio, IR = 1.36), PA (IR = 4.28), EA (IR = 10.33), and MG (IR = 4.79) are essential effectors in SR, it is noted that the amount of these four compounds increased during CSF, while cathechins decreased significantly, including cathechin (IR = 0.04) and EGC (IR = 0.02).

Although tannins in SR were reported to exhibit a myriad of pharmacological effects, the ability of tannins to exert such health effects mainly depends on their absorption and bioavailability. In fact, only 5–10% of the ingested polyphenols are estimated to be absorbed in their entirety by the small intestine (Bell et al., 2000). The absorption of tannins is largely influenced by the structures of these compounds. Only aglycones and a few glycosides can be absorbed in the intestinal mucosa. Studies suggested that enzymatic digestion of HTs to monomers (e.g., GA or EA) is able to further promote tannins' absorption in the stomach or small intestine. The gallotannins yield GA and glucose, owing to the esterase and depsidase activities of bacterial enzymes, while the ellagitannins mostly undergo lactonization to produce EA *in vivo* (Sallam et al., 2021). Urolithins, after further bacterial metabolism of EA in the colon, can be easily absorbed (Espin et al., 2013). HTs' monomers seem to have a better absorption and better effect compared to intact HTs. Blackberries and raspberries, for example, are good sources of EA at 1,500 ppm (Roche et al., 2017) and have a preventing effect against mammary tumors induced by estrogen. However, tumor volumes and multiplicity of rats consuming 40-ppm EA diet decreased significantly, while that of rats consuming blackberry or raspberry powder diet had a weaker reduction, demonstrating that EA was more effective than ellagitannins in berries. This shows that the absorption of the EA released from ellagitannins in gut flora is an essential factor in its efficiency. Meanwhile, weight loss was also prevented by using EA supplementation (Aiyer et al., 2008). Besides, flavan-3-ol monomers can also be fully absorbed from the small intestine. Several studies showed that catechin and epicatechin were rapidly absorbed from the upper portion of the small intestine (Bell et al., 2000), while the absorption rate of intact catechin dimers was measured to be 5–10% that of epicatechin monomers (Keqin and Liwei, 2014). Catechin dimers, trimers, and tetramers have a decreased rate of absorption, concurrent with increasing molecular size and the number of hydrophilic hydroxyl groups (Sallam et al., 2021).

Moreover, the hemostatic effects of phenolic acid have been widely reported, including those of GA, protocatechuic acid (Lin et al., 2014), and ferulic acid (Zhao et al., 2016), which all showed a significant increase in content in this study. Besides, EA is widely used in coagulation testing because it can initiate the endogenous coagulation process. Its methyl derivatives like 3,3′,4′-trimethylellagic acid can promote thrombocytopoiesis (Li et al., 2021). The contents of EA, 3,3′,4′-tri-O-methylellagic acid, and 3,4′-O-dimethylellagic acid, increased to 10.33 times, 3.17 times, and 6.61 times after CSF in this study, respectively. Phenolic acids released by tannins during CSF may contribute to CSR's hemostatic effect.

In summary, tannins exhibit moderate absorption in general, while their relevant monomers have a higher bioavailability, suggesting hydrolysis with released monomers is the key for absorption of dietary polyphenols *in vivo*. Such effective strategy could be implemented for SR by carbonizing via stir-frying, a common and traditional processing method for TCM in China. On the other hand, phenolic acids in CSR, which were produced in high amounts during CSF, have significant biological effects including hemostatic effect. Results from the study here provide more information to fill the gap between the chemical structures of tannins and the potential mechanism of SR in general practice, thereby facilitating discovery of the carbonizing mechanism to improve the efficacy of the medicinal materials.

## Conclusion

Mass spectrometry was used to investigate the effect of CSF on tannins transformation in SR. Qualitative analysis result conducted by UPLC-Q/TOF-MS/MS showed that the content level of tannins in SR decreased significantly after CSF, while its three categories, gallotannins, ellagitannins, and procyanidins, had downward trends in general. Content changes in HTs, including gallotannins and ellagitannins, suggested hydrolysis during CSF yielded GA and EA and their derivatives. Gallic, ellagic acid can further polymerize to form sanguisorbic acid dilactone. PCs, the oligomers of catechin, including procyanidin, procyanidin C2, procyanidin B3 and 3-O-galloylprocyanidin B3, decreased to form catechin and its derivatives, which may further be degraded to form PCA. Quantitative analysis by UHPLC-MS/MS illustrated that the amount of GA, PA, EA, and MG, the essential effectors in SR, increased significantly after CSF, and the contents of cathechin and EGC decreased remarkably. Tannins exhibit moderate absorption, while their relevant monomers have a higher bioavailability. Therefore, CSF is proved here to be an effective technique for the release of active monomers from the original polyphenol precursor. Results from this study explored the mechanism by which tannins are transformed upon CSF of SR.

## Data Availability

The original contributions presented in the study are included in the article/Supplementary Material, further inquiries can be directed to the corresponding authors.

## Abbreviations

CSF, carbonizing by stir-frying; CSR, charred *Sanguisorbae Radix*; EA, ellagic acid; ECG, epicatechin gallate; EG, ethyl gallate; EGC, epigallocatechin; FC, fold change; GA, gallic acid; HTs, hydrolyzable tannins; IR, increased ratio; MG, methyl gallate; NIs, the normalized intensities; PCs, proanthocyanidins; PA, pyrogallic acid; PCA, protocatechualdehyde; SR, *Sanguisorbae Radix*; TCM, traditional Chinese medicine.
