# Supplementary material for: Investigation of Tannins Transformation in Sanguisorbae Radix Over Carbonizing by Stir-Frying
Source: Front Mol Biosci. 2022 Mar 2;9:762224. doi: 10.3389/fmolb.2022.762224 (PMC8924296; doi:10.3389/fmolb.2022.762224)
Supplement: Supplementary file 1 [file Table1.docx]

Supplementary Table 1 Information of chemical constituents from SR/CSR(n=3)

| **No.** | **t_R_ (min)** | **Compounds** | **Chemical Formula** | **CAS No.** | **MW (Da)** | **m/z** | **MS^2^** | **Relative contents (%)** | | **Trend after charring** | **Polarity** |
| --- | --- | --- | --- | --- | --- | --- | --- | --- | --- | --- | --- |
|  |  |  |  |  |  |  |  | **SR** | **CSR** |  |  |
| 1 | 9.28 | 1,2,3,6-Tetragalloylglucose | C_34_H_28_O_22_ | 79886-50-3 | 788.1072 | 787.1064 | 169.0154(C_7_H_5_O_5_) | 0.395±0.124 | 0.056±0.002 | ↓↓ | [M-H]^-^ |
| 2 | 10.02 | 1,2,3,4,6-Pentagalloyl-β-D-glucose | C_41_H_32_O_26_ | 14937-32-7 | 940.1182 | 939.1169 | 169.0154(C_7_H_5_O_5_) | 0.214±0.085 | 0.031±0.012 | ↓↓ | [M-H]^-^ |
| 3 | 0.79 | 1-Galloyl-β-glucose | C_13_H_16_O_10_ | 13405-60-2 | 332.0743 | 331.0687 | 301.0589(C_12_H_13_O_9_)  169.0158(C_7_H_5_O_5_)  112.9867(C_4_HO_4_) | 1.548±0.407 | 0.269±0.007 | ↓↓ | [M-H]^-^ |
| 4 | 7.25 | Methoxybenzoic acid methyl ester-5-O-sulfate | C_9_H_10_O_8_S | - | 278.0096 | 277.0040 | 197.0464(C_9_H_9_O_5_)  179.0357(C_9_H_7_O_4_)  153.9752(C_7_H_3_O_4_) | 24.683±3.893 | 4.521±3.702 | ↓↓ | [M-H]^-^ |
| 5 | 7.80 | 1,3,6-tris(3,4,5-trihydroxybenzoate)-β-D-Glucopyranose | C_27_H_24_O_18_ | 18483-17-5 | 636.0963 | 635.0880 | 465.0100(C_20_H_17_O_13_) | 0.246±0.091 | 0.054±0.009 | ↓ | [M-H]^-^ |
| 6 | 6.33 | Gallic acid 3-O-β-D-(6'-O-galloyl)-glucopyranoside | C_20_H_20_O_14_ | 87087-61-4 | 484.0853 | 483.0810 | 321.0274(C_14_H_9_O_9_)  307.0118(C_13_H_7_O_9_) | 0.611±0.17 | 0.179±0.057 | ↓ | [M-H]^-^ |
| 7 | 5.59 | Chlorogenic acid | C_16_H_18_O_9_ | 327-97-9 | 354.0951 | 353.0779 | 335.0798(C_16_H_15_O_8_)  289.0743(C_15_H_13_O_6_） | 0.102±0.076 | 0.033±0.012 | ↓ | [M-H]^-^ |
| 8 | 2.64 | Methyl 6-O-galloyl-β-D-glucopyranoside | C_14_H_18_O_10_ | 88847-06-7 | 346.0900 | 345.0847 | 169.0124(C_7_H_5_O_5_)  112.9868(C_4_HO_4_) | 6.569±1.05 | 3.081±1.438 | ↓ | [M-H]^-^ |
| 9 | 5.22 | Glucosyringic acid | C_15_H_20_O_10_ | 33228-65-8 | 360.0156 | 359.0996 | - | 0.09±0.039 | 0.047±0.014 | ↓ | [M-H]^-^ |
| 10 | 7.43 | Ethyl gallate* | C_9_H_10_O_5_ | 831-61-8 | 198.1730 | 197.0469 | 112.9864(C_4_HO_4_) | 4.262±1.302 | 2.858±0.864 | ↓ | [M-H]^-^ |
| 11 | 6.70 | Methyl-6-O-digalloyl-β-D-glucopyranoside/Methyl-4,6-di-O-galloyl-β-D-glucopyranoside | C_21_H_22_O_14_ | - | 498.1010 | 497.0949 | - | 0.383±0.149 | 0.418±0.112 | → | [M-H]^-^ |
| 12 | 4.48 | β-1-O-galloyl-2,3-(S)-hexahydroxydiphenoyl-D-glucose | C_27_H_22_O_18_ | 84316-77-8 | 634.0806 | 633.0747 | 321.0281(C_14_H_9_O_9_)  169.0152(C_7_H_5_O_5_) | 0.122±0.058 | 0.161±0.064 | → | [M-H]^-^ |
| 13 | 7.06 | Brevifolincarboxylic acid | C_13_H_8_O_8_ | 18490-95-4 | 292.0219 | 291.0161 | 112.9900(C_4_HO_4_)  191.04(C_10_H_7_O_4_) | 0.088±0.032 | 0.121±0.039 | → | [M-H]^-^ |
| 14 | 1.16 | Gallic acid* | C_7_H_6_O_5_ | 149-91-7 | 170.0215 | 171.0288 | 153.0182(C_7_H_5_O_4_)  127.0389(C_6_H_7_O_3_)  112.9865(C_4_HO_4_） | 4.618±1.952 | 8.309±1.830 | ↑ | [M+H]^+^ |
| 15 | 5.23 | [Protocatechuic acid](https://www.chemsrc.com/en/cas/99-50-3_1188518.html) | C_7_H_6_O_4_ | 99-50-3 | 154.0226 | 153.0199 | 112.9867(C_4_HO_4_) | 0.01±0.007 | 0.083±0.02 | ↑↑ | [M-H]^-^ |
| 16 | 8.54 | Ferulic Acid | C_10_H_10_O_4_ | 1135-24-6 | 194.0579 | 193.0521 | 112.9866(C_4_HO_4_)  163.0411(C_9_H_7_O_3_) | 0.004±0.004 | 0.051±0.01 | ↑↑ | [M-H]^-^ |
| 17 | 1.90 | Ethyl 3,4-dihydroxybenzoate | C_9_H_10_O_4_ | 3943-89-3 | 182.0579 | 180.9743 | 125.0264(C_6_H_5_O_3_)  153.0538(C_8_H_9_O_3_)  169.0146(C_8_H_9_O_4_) | 0.007±0.003 | 0.09±0.004 | ↑↑ | [M-H]^-^ |
| 18 | 10.75 | [Methyl 3,4-methoxy-5-hydroxybenzoate](https://www.chemsrc.com/en/cas/83011-43-2_27689.html) | C_10_H_12_O_5_ | 83011-43-2 | 212.0685 | 213.0757 | 181.0493(C_9_H_9_O_4_) | 0.075±0.023 | 1.259±0.277 | ↑↑ | [M+H]+ |
| 19 | 1.53 | Pyrogallic acid | C_6_H_6_O_3_ | 87-66-1 | 126.0317 | 125.0253 | - | 0.301±0.075 | 5.579±1.509 | ↑↑ | [M-H]^-^ |
| 20 | 8.17 | 3-O-galloylnorbergerin | C_20_H_18_O_13_ | - | 466.0747 | 465.0704 | - | 0.015±0.011 | 0.332±0.064 | ↑↑ | [M-H]^-^ |
| 21 | 4.11 | Methyl gallate* | C_8_H_8_O_5_ | 99-24-1 | 184.1460 | 183.0311 | 119.0369(C_4_H_7_O_4_)  112.9867(C_4_HO_4_) | 0.042±0.031 | 5.483±1.343 | ↑↑ | [M-H]^-^ |
| 22 | 13.34 | 3,3',4'-trimethylellagic acid-4-O-sulphate | C_17_H_12_O_11_S | - | 424.0100 | 423.0758 | 408.9888(C_16_H_9_O_11_S)  343.0464(C_17_H_11_O_8_)  248.9620(C_14_HO_3_S)  112.9867(C_4_HO_4_) | 6.348±2.322 | 0.013±0.008 | ↓↓ | [M-H]^-^ |
| 23 | 12.60 | 3,4'-dimethylellagic acid-4-O-sulphate | C_16_H_10_O_11_S | - | 409.9944 | 408.9873 | 329.0326(C_16_H_9_O_8_)  248.9622(C_14_HO_3_S) 112.9868(C_4_HO_4_) | 7.781±1.267 | 0.198±0.15 | ↓↓ | [M-H]^-^ |
| 24 | 12.23 | 3,4'-di-O-methylellagic acid-4-O-β-D-xylopyranoside | C_21_H_18_O_12_ | 62218-23-9 | 462.0798 | 461.0762 | 112.9870(C_4_HO_4_) | 0.384±0.114 | 0.03±0.008 | ↓↓ | [M-H]^-^ |
| 25 | 4.48 | 3,3',4'-tri-O-methylellagic acid-4-O-β-D-xylopyranoside | C_22_H_20_O_12_ | 136133-08-9 | 476.0955 | 475.1057 | 361.0720(C_21_H_17_O_12_)  321.0252(C_14_H_9_O_9_) | 0.093±0.045 | 0.027±0.013 | ↓ | [M-H]^-^ |
| 26 | 8.91 | Ellagic acid 4-O-xylopyranoside | C_19_H_14_O_12_ | 139163-18-1 | 434.0485 | 433.0447 | 359.0081(C_16_H_7_O_10_)  112.9868(C_4_HO_4_) | 0.028±0.016 | 0.065±0.031 | ↑ | [M-H]^-^ |
| 27 | 15.55 | 3,3',4'-tri-O-methylellagic acid | C_17_H_12_O_8_ | 1617-49-8 | 344.0532 | 343.0480 | 300.9020(C_14_H_5_O_8_)  112.9866(C_4_HO_4_) | 0.035±0.014 | 0.111±0.031 | ↑ | [M-H]^-^ |
| 28 | 12.60 | 3,4’-O-dimethylellagic acid | C_16_H_10_O_8_ | 57499-59-9 | 330.0376 | 329.0321 | - | 0.083±0.024 | 0.549±0.114 | ↑↑ | [M-H]^-^ |
| 29 | 9.28 | Ellagic acid* | C_14_H_6_O_8_ | 476-66-4 | 302.0063 | 301.0010 | 137.0253(C_7_H_5_O_3_)  112.9865(C_4_HO_4_) | 0.392±0.118 | 3.015±0.487 | ↑↑ | [M-H]^-^ |
| 30 | 10.75 | Sanguisorbic acid dilactone | C_21_H_10_O_13_ | 82203-11-0 | 470.0121 | 469.0071 | 300.9996(C_14_H_5_O_8_) | 0.015±0.009 | 0.602±0.079 | ↑↑ | [M-H]^-^ |
| 31 | 2.27 | (-)-Gallocatechin | C_15_H_14_O_7_ | 3371-27-5 | 306.0740 | 307.0812 | 167.0128(C_11_H_3_O_2_)  135.0441(C_8_H_7_O_2_)  113.0597(C_6_H_9_O_2_) | 0.180±0.080 | 0.000±0.000 | ↓↓ | [M+H]+ |
| 32 | 5.22 | (-)-Epigallocatechin | C_15_H_14_O_7_ | 970-74-1 | 306.0740 | 307.0812 | - | 0.113±0.024 | 0.000±0.000 | ↓↓ | [M+H]+ |
| 33 | 5.22 | Procyanidin B3 (Catechin dimer) | C_30_H_26_O_12_ | 23567-23-9 | 578.1424 | 577.1359 | 289.0743(C_15_H_13_O_6_)  305.0682(C_15_H_13_O_7_) | 3.312±0.764 | 0.232±0.131 | ↓↓ | [M-H]^-^ |
| 34 | 8.17 | 3-O-galloylprocyanidin B3 (Derivative of catechin dimer) | C_37_H_30_O_16_ | 73086-04-1 | 730.1534 | 729.1497 | 577.1375(C_30_H_25_O_12_) | 5.181±1.484 | 0.380±0.201 | ↓↓ | [M-H]^-^ |
| 35 | 3.01 | Procyanidin | C_30_H_26_O_13_ | 4852-22-6 | 594.1373 | 593.1330 | 345.0864(C_16_H_9_O_9_)  313.0582(C_13_H_13_O_9_)  112.9863(C_4_HO_4_) | 0.468±0.173 | 0.036±0.026 | ↓↓ | [M-H]^-^ |
| 36 | 5.59 | Procyanidin C2 (Catechin trimer) | C_45_H_38_O_18_ | 37064-31-6 | 866.2058 | 865.2011 | 579.1542(C_30_H_27_O_12_)  357.0616(C_18_H_13_O_8_)  325.0508(C_21_H_9_O_4_)  289.0743(C_15_H_13_O_6_) | 0.435±0.152 | 0.041±0.032 | ↓↓ | [M-H]^-^ |
| 37 | 5.59 | Catechin* | C_15_H_14_O_6_ | 154-23-4 | 290.2680 | 289.0736 | 193.0104(C_9_H_5_O_5_)  165.0183(C_8_H_5_O_4_)  137.0252(C_7_H_5_O_3_)  112.9867(C_4_HO_4_) | 9.674±1.416 | 1.100±0.573 | ↓↓ | [M-H]^-^ |
| 38 | 7.80 | Fisetinidol (4β→8)-catechin | C_30_H_26_O_11_ | [57526-59-7](https://www.chemsrc.com/baike/244844.html) | 562.1475 | 561.1439 | 317.0430(C_17_H_17_O_6_)  289.0713(C_15_H_13_O_6_)  227.0047(C_5_H_7_O_10_) | 0.315±0.153 | 0.053±0.017 | ↓↓ | [M-H]^-^ |
| 39 | 9.28 | [(-)-Epicatechin gallate](https://www.chemsrc.com/en/cas/1257-08-5_598585.html) | C_22_H_18_O_10_ | [1257-08-5](https://www.chemsrc.com/baike/598585.html) | 442.0900 | 441.0846 | 287.0564(C_15_H_11_O_6_)  169.0151(C_7_H_5_O_5_)  112.9865(C_4_HO_4_) | 0.144±0.048 | 0.033±0.006 | ↓ | [M-H]^-^ |
| 40 | 3.38 | Protocatechualdehyde | C_7_H_6_O_3_ | 139-85-5 | 138.0317 | 137.0251 | 112.9864(C_4_HO_4_) | 0.105±0.014 | 2.084±0.812 | ↑↑ | [M-H]^-^ |

* marked after compound name means mass spectrum of the compound has been identified with that of corresponding standard.

“↑” means FC≥1.5, and “↑↑” means FC≥5. “↓” means FC≤0.67, and “↓↓” means FC≤0.2. “→” means 0.67<FC<1.5.
